# Fungal endophyte-induced salidroside and tyrosol biosynthesis combined with signal cross-talk and the mechanism of enzyme gene expression in *Rhodiola crenulata*

**DOI:** 10.1038/s41598-017-12895-2

**Published:** 2017-10-02

**Authors:** Jin-Long Cui, Ya-Nan Wang, Jin Jiao, Yi Gong, Jun-Hong Wang, Meng-Liang Wang

**Affiliations:** 1 Institute of Applied Chemistry, Shanxi University, Taiyuan, 030006 China; 2 Institute of Biotechnology, Shanxi University, Taiyuan, 030006 China

## Abstract

Endophyte is a factor that affects the physiology and metabolism of plant. However, limited information is available on the mechanism of interaction between endophyte and plant. To investigate the effects of endophytic fungus ZPRs-R11, that is, *Trimmatostroma* sp., on salidroside and tyrosol accumulations in *Rhodiola crenulata*, signal transduction, enzyme gene expression, and metabolic pathway were investigated. Results showed that hydrogen peroxide (H_2_O_2_), nitric oxide (NO), and salicylic acid (SA) involved in fungus-induced salidroside and tyrosol accumulations. NO acted as an upstream signal of H_2_O_2_ and SA. No up- or down-stream relationship was observed, but mutual coordination existed between H_2_O_2_ and SA. Rate-limiting enzyme genes with the maximum expression activities were UDP-glucosyltransferase, tyrosine decarboxylase (*TYDC*), monoamine oxidase, phenylalanine ammonialyase (*PAL*), and cinnamic-4-hydroxylase sequentially. Nevertheless, the genes of tyrosine transaminase and pyruvate decarboxylase only indicated slightly higher activities than those in control. Thus, TYDC and PAL branches were the preferential pathways in ZPRs-R11-induced salidroside and tyrosol accumulation. *Trimmatostroma* sp. was a potential fungus for promoting salidroside and tyrosol accumulations. The present data also provided scientific basis for understanding complex interaction between endophytic fungus and *R. crenulata*.

## Introduction

*Rhodiola crenulata* (HK. f. et. Thoms) H. Ohba is an alpine plant and a functional food and medicine with adaptogenic bioactivity, which is mostly attributed by its main ingredients, namely, salidroside (2-(4-hydroxyphenyl)ethyl-β-D-glucopyranoside) and tyrosol (4-(2-hydroxyethyl)phenol) (Supplementary Fig. S1)^1^. This plant is commonly eaten by people working in deep sea or high mountain and those sportsmen and astronauts^2^. The high cold regions at Qinghai–Tibet Plateau with altitude of more than 3,500 m are the main distribution areas of geo-authentic *R. crenulata* (Supplementary Fig. S1), which is known as “plateau ginseng” due to its pharmacological efficacy, such as antifatigue, antianoxia, antimicrowave radiation, and prolongation of life^1,3^. However, in recent years, the largely declined wild resource of *R. crenulata* is not easily addressed due to the slow growth and overexploitation of this plant^4^. Although people tried to introduce this plant artificially to areas at low altitude, the salidroside and tyrosol contents are relatively low in *R. crenulata* after growing 5–7 years^4,5^. Currently, guaranteeing salidroside and tyrosol contents in *R. crenulata* and how to improve their cultivation away from their native environment are both important topics. As an important influencing factor in plant growth, development, and secondary metabolite accumulation, endophytic fungi received considerable attention^6^.

Endophytes can establish symbiotic relationship with host plant, but they will not cause evident symptoms and strong hypersensitive reaction^6,7^; as an important environmental constituent of healthy plant, endophytes can largely influence the secondary metabolite profile of the host plant^7^. With the invasion of endophytic fungus, a series of events will occur; these events include recognition to fungus; release of reactive oxygen species (ROS), such as hydrogen peroxide (H_2_O_2_); flow of ion, such as K^+^/H^+^ and Cl^−^/Ca^2+^; cross-talk of signal molecules, such as nitric oxide (NO); signal message integration by transcription factors (TFs); and up- or down-regulation of gene expression, thereby resulting in closing or opening of some special secondary metabolic pathways in the accumulation of desired defense secondary metabolites^8^.

Signal molecules mediate the interaction between endophyte and plant^8,9^. In a specific fungus-induced reaction, not all signals (pathways) are involved; they do not work alone, but they are in a cross-talk^10^. Knowledge about which and how signal molecules are involved in a special endophyte-induced secondary metabolite accumulation is largely limited. When the oxidation–reduction is not in equilibrium with the invasion of endophyte, oxidative stress would lead to ROS generation, which depends on NADPH or peroxidase^11^. H_2_O_2_ is a key signaling molecule in ROS that can activate and regulate the expression of defense genes, such as lipoxygenase pathway^12^. Nevertheless, H_2_O_2_ release is not always related with the accumulation of secondary metabolites. H_2_O_2_ is not involved in endophytic fungus-induced isoeuphpekinensin accumulation in *Euphorbia pekinensis* ^13^. NO is a common and key node of complicated transduction network in fungus-induced secondary metabolite accumulation^14^; NO is also the upstream signal molecule of other signals, such as salicylic acid (SA), H_2_O_2_, and methyl jasmonate^15^. Except for Ca^2+^, NO, H_2_O_2_, and SA are the first signals detected sequentially in endophytic *Gilmaniella* sp.; they regulate sesquiterpenoid production in *Atractylodes lancea* ^16^. SA is also a high frequency signaling molecule in response to biotic stress; notably, this molecule is involved in fungus-induced products^17^. However, SA is not present in all fungus-induced secondary metabolite accumulation in plant. SA treatment exerts no influence on betacyanin synthesis in *Amaranthus* seedlings under light or dark conditions^18^. Thus, whether and how the signals work in a special endophyte and plant need further scientific basis.

Signal transductions caused by endophyte invasion will be integrated by TF, and some gene expressions would be regulated to produce the desired secondary metabolites in host plant in response to defense reaction^8,9^. When *A*. *lancea* is infected with endophytic *Acinetobacter* sp., 3-hydroxy-3-methylglutaryl-CoA reductase and 1-deoxy-D-xylulose 5-phosphate reductoisomerase genes are up-regulated; these genes are related with mevalonate pathway in bacterium-induced sesquiterpenoid production^17^. In *Rhodiola* plant, the synthesis of salidroside and tyrosol originated from the cinnamic acid pathway. Nevertheless, the branch pathway that is preferential among phenylalanine ammonialyase (PAL), tyrosine aminotransferase (TAT), and tyrosine decarboxylase (TYDC) branch pathways is still unknown^19,20^. The enzyme gene expression and metabolism of salidroside and tyrosol in *R. crenulata* affected with endophytic fungus are also unclear. Further investigation should be performed to understand the complex synthesis mechanism of fungus-induced salidroside and tyrosol in *R. crenulata* ^19^.

Although many events are involved in endophytic fungus-induced secondary metabolite accumulation in plant^8^, the molecular mechanism mediating secondary metabolite production, especially in rare functional plants, such as *R. crenulata*, has not been investigated widely. In the present study, several bioactive endophytic fungi, such as *Lachnum* sp. (Rac12)^21^, *Phialocephala fortinii* (ZPRa-R-1)^22^, and *Trimmatostroma* sp. (ZPRs-R11), were obtained from *Rhodiola* spp^21^., which could grow healthily with *R. crenulata* plantlet and promote salidroside and tyrosol accumulations. In this paper, we report the function and interaction of NO, SA, and H_2_O_2_ and the key enzyme gene expression activity in synthesis pathway of salidroside and tyrosol in *R. crenulata* plantlet, as induced by endophytic *Trimmatostroma* sp. Furthermore, we revealed the mechanism of signal transduction and biosynthesis branch pathway of salidroside and tyrosol.

## Results

### Endophytid fungus-induced salidroside and tyrosol accumulations

**Figure 1:**
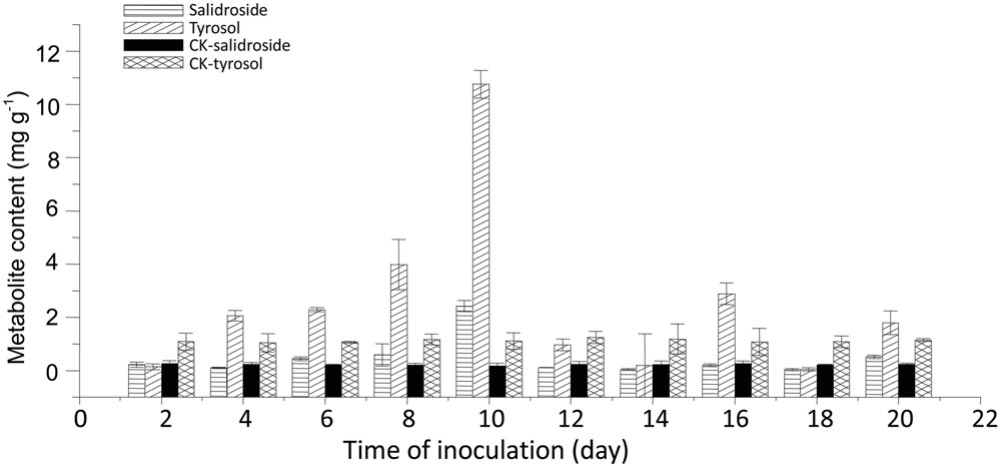
Effects of endophytic fungus ZPRs-R11 (*Trimmatostroma* sp.) on salidroside and tyrosol accumulations in *R. crenulata* plantlets in a test cycle of 20 days. The endophytic fungus-induced salidroside and tyrosol contents reached 2.424 and 10.759 mg g^−1^ on the 10th day, which were13.7- and 9.7-fold those of the control, respectively. Data are represented with means ± standard deviation (SD) to three replicates by Microsoft.

According to the quantitative results of ^1^H-NMR, UPLC-Q/TOF/-MS, and HPLC, the endophyte ZPRs-R11 could promote salidroside and tyrosol accumulations. Figure 1 shows the time course of salidroside and tyrosol accumulations in *R. crenulata* inoculated with ZPRs-R11. The maximum contents of salidroside and tyrosol reached 2.424 and 10.759 mg g^−1^ on the 10th day of fungus-inoculated plantlet, and they were 13.7- and 9.7-fold of those of the controls, respectively. The symbiosis relationship was verified through microtome investigation (Supplementary Fig. S1). Endophytic fungus was re-isolated and re-identified through comparison of its r-DNA ITS sequence.

### Involvement of H_2_O_2_, NO, and SA signaling pathways

**Figure 2:**
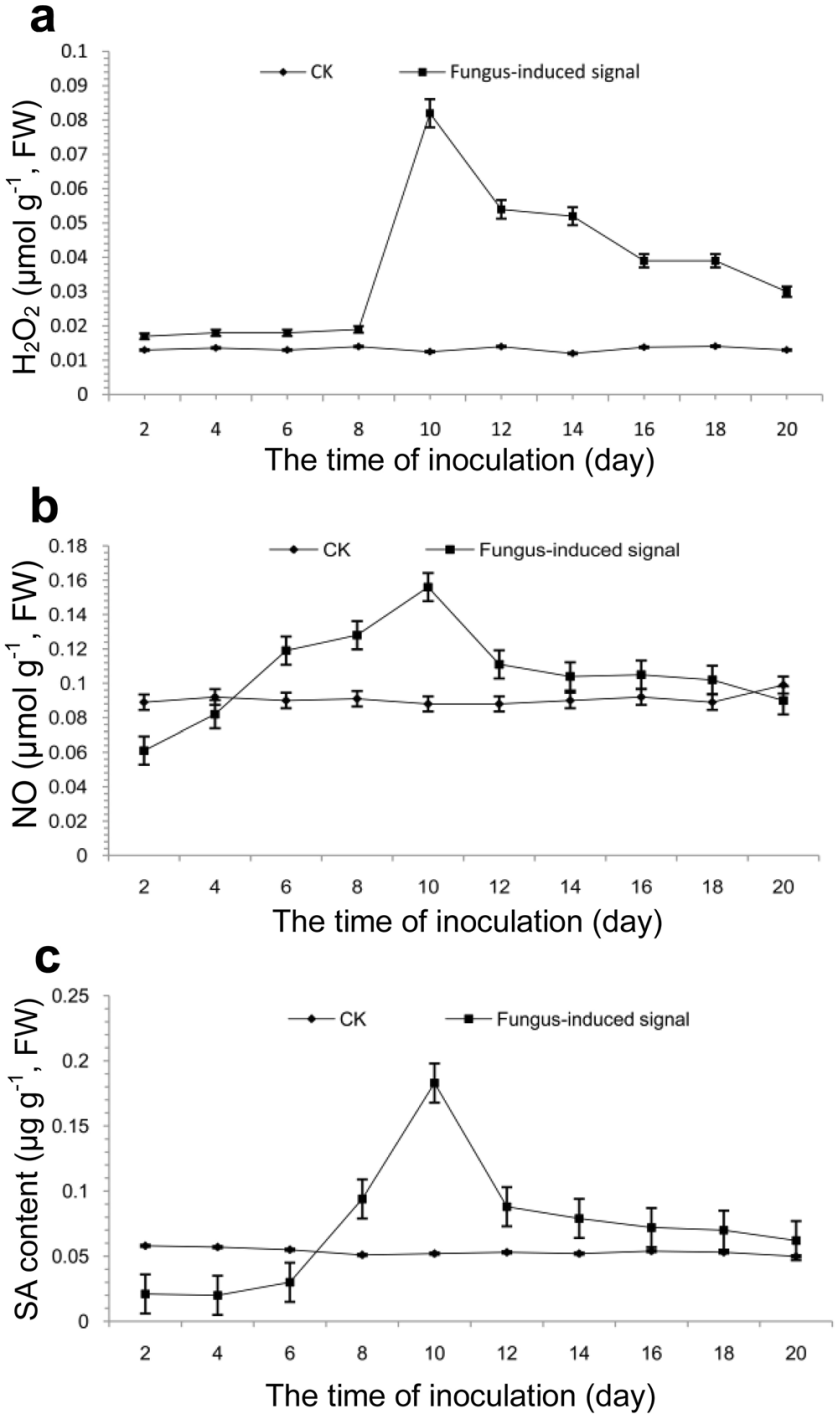
Time course of signals, including hydrogen peroxide (H_2_O_2_) (**a**), nitric oxide (NO) (**b**), and salicylic acid (SA) (**c**), produced in *R. crenulata* plantlets inoculated with endophytic fungus ZPRs-R11 (*Trimmatostroma* sp.) in a test cycle of 20 days. The maximum contents of H_2_O_2_, NO, and SA could reach 0.082 μmol g^−1^, 0.156 μmol g^−1^, and 0.183 μg g^−1^, which were 6.56-, 1.77-, and 3.52-fold those of controls on the 10th day, respectively. Results are presented as the means ± SD of three replicates. *P < 0.05, **P < 0.01, statistically significant in comparison with the control. FW: fresh weight.

Detection results showed that H_2_O_2_, NO, and SA were involved the accumulation of ZPRs-R11-induced salidroside and tyrosol. As indicated in Fig. 2, higher levels of H_2_O_2_, NO, and SA were observed in ZPRs-R11 treatment than those in control, which exhibited that the endophytic fungus might trigger the biosynthesis of H_2_O_2_, NO, and SA in *R. crenulata*. The maximum contents of H_2_O_2_, NO, and SA could reach 0.082 μmol g^−1^, 0.156 μmol g^−1^, and 0.183 μg g^−1^, and they were 6.56-, 1.77-, and 3.52-fold of those in controls on the 10th day, respectively.

### Dependence of fungus-induced metabolite accumulation on H_2_O_2_

**Figure 3:**
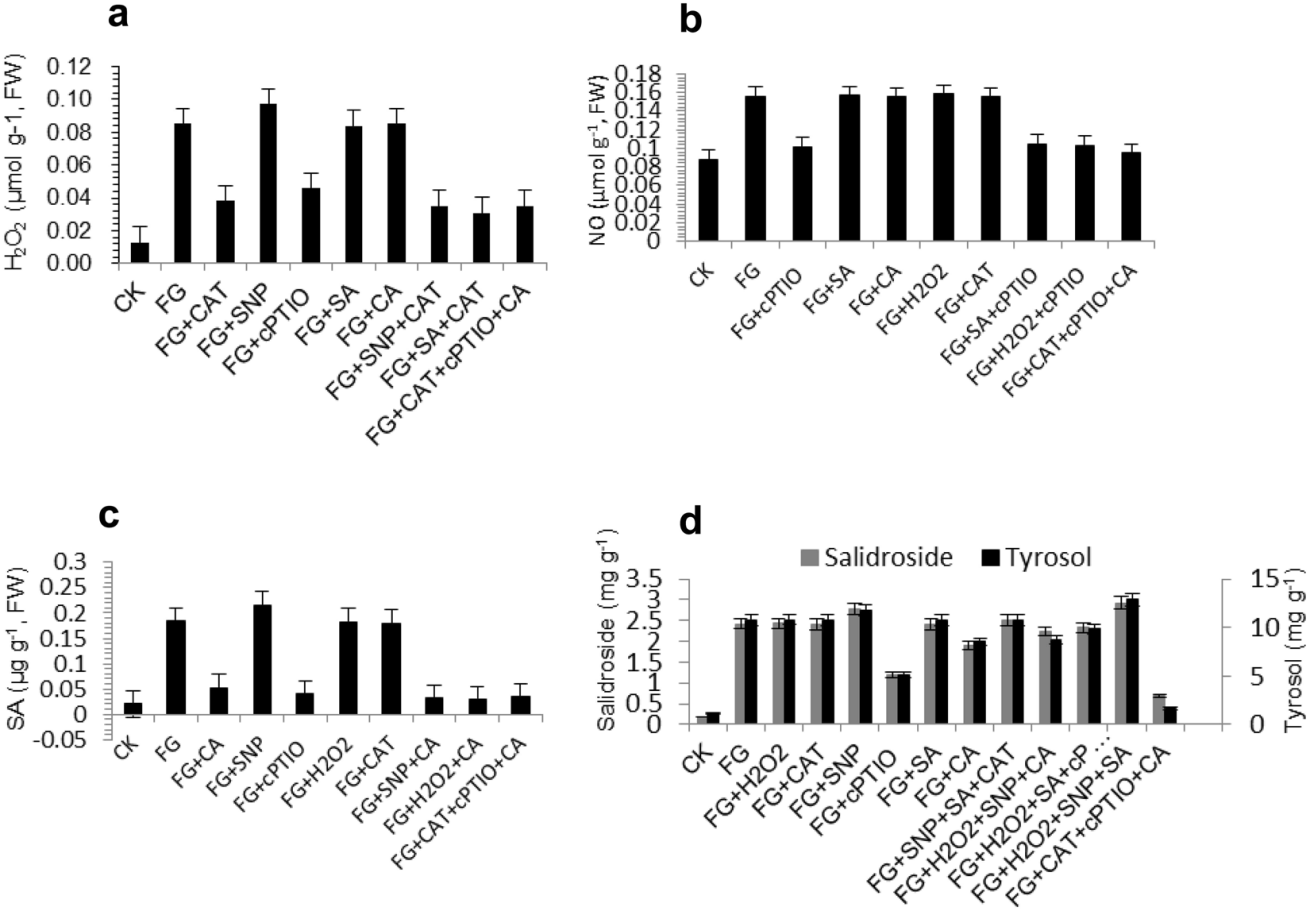
Interaction relationships among H_2_O_2_, NO, and SA (**a**,**b**, and **c**) and the effects of these signals on fungus-induced salidroside and tyrosol accumulations (**d**) in *R. crenulata* plantlets inoculated with endophytic fungus ZPRs-R11. NO was a key node signal molecule. NO and SA exhibited remarkable activities in accumulation of the two metabolites. The working concentration of SNP, SA, H_2_O_2_, and CA was 1 mM, and the cPTIO and CAT concentrations were 30 μM and 2 mkat L^−1^, respectively. SNP: sodium nitroprusside; SA: salicylic acid, H_2_O_2_: hydrogen peroxide; CA: trans-cinnamic acid; cPTIO: 2-(4-carboxyphenyl)-4,4,5,5-tetramethylimidazoline-1-oxyl-3-oxide potassium salt; CAT: catalase; FW: fresh weight. Plantlets were treated on the 10th day. Values were the means of three repeats ± standard error.

As shown in Fig. 3a, ZPRs-R11 could significantly induce H_2_O_2_ accumulation (0.085 μmol g^−1^) compared with that of control (0.013 μmol g^−1^). Nevertheless, H_2_O_2_ accumulation was significantly suppressed to 0.038 μmol g^−1^ by CAT (2 mkat L^−1^) on the 10th day after inoculation. SNP could enhance H_2_O_2_ accumulation (0.097 μmol g^−1^), and its scavenger cPTIO could significantly inhibit at 0.046 μmol g^−1^ of H_2_O_2_ triggered with ZPRs-R11. The donor (SA) and inhibitor (CA) of SA exerted no remarkable effects on H_2_O_2_ accumulation. CAT could inhibit the promotion effects of exogenous SNP and SA in *R. crenulata* infected with ZPRs-R11. However, CAT, cPTIO, and CA were not the only factors suppressing H_2_O_2_ production because combined CAT, cPTIO, and CA could not completely inhibit the H_2_O_2_ content to the control level in plantlets inoculated with ZPRs-R11. The contents of ZPRs-R11-induced salidroside and tyrosol could slightly increase to 10.802 and 2.435 mg g^−1^ by adding exogenous H_2_O_2_ (1 mM) (Fig. 3d). Nonetheless, the chemical contents of the two metabolites were not evidently affected by inhibitor CAT (Fig. 3d). All these results indicated that H_2_O_2_ was an essential signal, but it played limited role in fungus-induced salidroside and tyrosol accumulations.

### NO as a necessary signal in fungus-induced product accumulation

NO content increased from 0.088 μmol g^−1^ to 0.156 μmol g^−1^ in *R. crenulata* inoculated with ZPRs-R11 on the 10th day. Figure 3b shows that the scavenger cPTIO (30 μM) could considerably inhibit the NO accumulation triggered by endophytic fungus ZPRs-R11. However, both CAT (2 mkat L^−1^) and CA (1 mM) could not suppress NO accumulation, and their H_2_O_2_ (1 mM) and SA (1 mM) donors could not also promote NO to obtain higher yield than that of plantlet inoculated with ZPRs-R11. These results indicated that NO was the upstream signal of H_2_O_2_ and SA. Figure 3d illustrates that exogenous NO (1 mΜ SNP) exhibited its advantage in promoting fungus-induced salidroside (2.784 mg g^−1^) and tyrosol (11.768 mg g^−1^) accumulations. In addition, cPTIO prevented these metabolites from accumulation induced by ZPRs-R11. These results showed that NO played a vital role in fungus-induced salidroside and tyrosol accumulations, and it was a necessary signaling pathway.

### Involvement of fungus-induced metabolite accumulation on SA

Figure 3c shows that CA, the inhibitor of SA, can decrease SA content from 0.184 μg g^−1^ to 0.053 μg g^−1^ in *R. crenulata* triggered with ZPRs-R11 on the 10th day. As the NO donor, SNP (1 mM) could promote SA content to increase slightly to 0.216 μg g^−1^. cPTIO (30 μM) largely suppressed fungus-induced SA accumulation, which showed that SA was a downstream signal molecule of NO. The other signal molecule H_2_O_2_ and its inhibitor could slightly affect SA accumulation. Figure 3d illustrates that the contents of fungus-induced salidroside and tyrosol increased slightly, which was related with SA (1 mM). On one hand, CA could decrease the fungus-induced salidroside and tyrosol from 2.424 mg g^−1^ to 1.895 mg g^−1^ and 10.759 mg g^−1^ to 8.524 mg g^−1^, respectively. On the other hand, cPTIO and CAT could not suppress the function of SA-induced salidroside and tyrosol accumulations in *R. crenulata* inoculated with ZPRs-R11. These results showed that SA was an important signaling pathway in fungus-induced salidroside and tyrosol accumulations.

### Cross-talk of H_2_O_2_, NO, and SA in synthesis pathways

**Figure 4:**
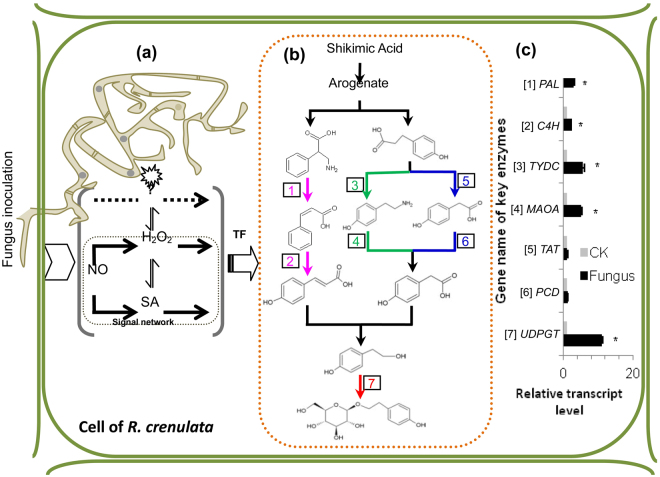
Proposed signal network, synthetic metabolic pathway, and the key enzyme activities of fungus-induced salidroside and tyrosol in *R. crenulata* plantlets inoculated with ZPRs-R11 on the 10th day. With the invasion of fungus ZPRs-R11, NO, H_2_O_2_, and SA were involved in transmitting signals, which were integrated by transcription factors (**a**), which could activate the synergic expression of multiple genes and activate the expression of genes regulating similar secondary metabolites, such as salidroside and tyrosol (**b**,**c**)^8^. Results showed that [3] tyrosine decarboxylase gene (*TYDC*) and [4] monoamine oxidase gene (*MAOA*) exhibited the highest relative transcript level, followed by that of [1] phenylalanine ammonialyase gene (*PAL*), [2] cinnamic-4-hydroxylase gene (*C4H*), [5] tyrosine transaminase gene (*TAT*), and [6] pyruvate decarboxylase gene (*PCD*). Such result indicated that TYDC and PAL branch pathways played more vital role than that of TAT branch pathway in *R. crenulata* plantlets inoculated with ZPRs-R11. The relative expression levels were evaluated using 2^−ΔΔCT^ method^40^. Data were represented with means ± SD (n = 3) and normalized against the double-reference gene phytochelatin synthase (*PCS*) and ubiquitin (*UBQ*). Bars with asterisks denote significant difference from the control (*P < 0.05).

Figure 2 shows that the contents of three signals, namely, H_2_O_2_, NO, and SA, could be increased by inducing endophytic fungus ZPRs-R11 in *R. crenulata*. However, combined CAT, cPTIO, and CA only could partly suppress H_2_O_2_, NO, and SA production. This observation showed that these three signals were only a small part in the signal network, which was also affected with other factors (Fig. 3). cPTIO could suppress the increase in all of these three signals. Nevertheless, CAT could decrease H_2_O_2_ but partly decrease SA. CA could decrease SA but partly decrease H_2_O_2_. Both them could not suppress NO increase, which showed that NO was an upstream signal of H_2_O_2_ and SA. A mutual interaction occurred between H_2_O_2_ and SA, but no up- or down-stream relationship existed between them. As shown in Fig. 3, combined H_2_O_2_, SNP, and SA could promote fungus-induced salidroside and tyrosol accumulations, but their inhibitor or scavenger could not totally decrease the metabolite contents to the level of the control; this result showed that H_2_O_2_, NO, and SA were only parts of the overall signal network (Fig. 4a).

### Transcriptional responses of enzyme genes and synthesis branch pathway

As shown in Fig. 4, the *UDPGT* (⑦) gene expression level and salidroside content were considerably significantly correlated, and the Pearson correlation coefficient (PCC) was 0.996 (*P* < 0.01). The PCCs of *TYDC* (③) and *MAOA* (④) genes were 0.850 and 0.840, and those of *PAL* (①) and *C4H* (②) genes were 0.806 and 0.869, which showed significant correlation (*P* < 0.05). However, the PCCs of *TAT* (⑤) and *PCD* (⑥) were only 0.524 and 0.687. The relative expression levels of *UDPGT*, *TYDC*, *MAOA*, *PAL*, *C4H*, *TAT*, and *PCD* were 11.06-, 5.71-, 5.13-, 2.90-, 2.49-, 1.20-, and 1.26-fold of those of control levels, respectively. The salidroside and tyrosol contents were 13.69- and 9.67-fold of those of control in *R. crenulata* inoculated with ZPRs-R11 on the 10th day (Fig. 3). The presented results showed that ZPRs-R11 could promote tyrosol and salidroside accumulations, and TYDC and PAL branch pathways, followed by TAT branch pathway, showed the most contribution to the syntheses these metabolites (Fig. 4).

## Discussion

The opinion that fungal endophyte plants, as a fate community, live and respond together to the environmental stress in natural conditions is supported by many biologists^6^. Recently, scientists found that elicitor, such as oligosaccharide, polyunsaturated fatty acid, and chitosan from fungal endophyte cell, can also regulate secondary metabolite accumulation in host plant^9,23^. In our study, protein and amino acid, oligosaccharide, polysaccharide, lipid, and glycoprotein were isolated from the mycelium of ZPRs-R11 and its fermentation broth. Subsequently, their effects on salidroside and tyrosol accumulations in *R. crenulata* were investigated. Results indicated that only polysaccharide (1 mg/mL) slightly promoted salidroside and tyrosol accumulations by 1.27- and 1.62-fold of those in controls after six days of treatment. No results better than that of living fungus application could be obtained from any single elicitor component. Similar finding also indicated that endogenous fungal component as elicitor didn’t cause the oxidative increase and reactive ROS release in *E*. *pekinensis* cells^24^. Presumably, exceptions exist in complicated interaction between special endophytic fungus and plant. Living fungi exhibit several advantages over an elicitor: (i) fungi can grow continually with host growth and release metabolites or constantly interact with host tissue or cell, which can constantly trigger host defense response. (ii) Fungi can establish permanent symbiosis relationship with host without causing evident symptoms of infection. People need not to maintain the fungus after inoculating them. Several whole endophytic fungi can already be applied in agricultural production in large-scale commercialization. For example, modified *Neotyphodium lolii* (E^AR1^ and E^AR37^) is inoculated on *Lolium perenne* to improve the quality of feeding livestock^25^, and *Trichoderma harziamum*, *Paecilomyces lilicinus*, *Beauveria bassiana*, and *Fusarium oxysporum* are used as commercial biological control agents^26^.

Compared with millions of plant, only about dozens of species were investigated regarding the signal transduction interaction between endophytic fungus and host to date^8^. Among them, *Cupressus lusitanica*, *Catharanthus roseus*, and *Panax ginseng* are the most commonly researched plants about signal transduction^27,28^. In the signal network, many signal molecules, such as NO, IP_3_, O_2_^−^, ROS, H_2_O_2_, JA, SA, ABA, ethylene, Ca^2+^, cAMP, and G protein, are related with the secondary metabolite accumulation. NO, H_2_O_2_, JA, SA, and ROS are regarded as essential signaling molecules in the process of fungus-induced metabolite production^9^. NO can improve the whole lines of signal transduction^8^, and it is a key signal that can trigger many signals, such as H_2_O_2_ and ethylene increase^8^, and induce taxol^29^, ginseng saponin^30^, and catharanthine accumulation^31^. Considerable amount of evidence proved that mutual coordination is a common phenomenon among signals, such as Ca^2+^ and brassinolide^32^, and coordination among JA, ethylene, SA, H_2_O_2_, and NO results in sesquiterpenoid accumulation in *A*. *lancea* induced with endophytic fungus *Gilmaniella* sp^16^. However, further basis is needed to support this argument because of exception^13,18^. Thus, our results provided further understanding of signal transduction in fungus-induced secondary metabolites production.

Selection of suitable reference gene is important to reduce error on yield, quality, and efficiency of reverse transcription of desired gene mRNAs using qRT-PCR. Most often, glyceraldehydes-3-phosphate dehydrogenase (*GAPDH*), beta-actin (*ACT*), and elongation factor 1 alpha (*EF-1α*) are used as reference genes^33^. Nevertheless, the result would be different from the true value because no gene remains stable under different conditions^34^. Gutierrez *et al*.^34^ found that the expression levels of target gene showed 100-fold difference between validated and nonvalidated reference genes. Therefore, the reference genes should be carefully selected in a particular study, and the numbers should not be only one, as shown in the latest consensus^33^. For example, *UBI1* is selected as reference gene in *Rosa hybrid* treated with drought stress, and *ACT4* is suitable for *R. hybrid* treated with gibberellin or abscisic acid^35^. *GAPDH* combined with *EF-1α* is a double-reference-gene used in the study of the reproductive stage of *Jatropha curcas* ^36^. In the present study, the expression levels of *PCS* and *UBQ* were stable, as evaluated by geNorm, Normfinder, and BestKeeper software. *PCS* combined with *UBQ* as a double-reference-gene could obtain precise results on the gene expression in *R. crenulata* plantlet inoculated with *Trimmatostroma* sp., which is a novel discovery.

The synthesis pathway of salidroside and tyrosol was mainly based on TYDC and PAL branch pathways sequentially in *R. crenulata* induced with *Trimmatostroma* sp. (Fig. 4). In *Phialocephala* sp., PAL and TYDC branch pathways sequentially are the most prioritized branch in the synthesis of fungus-induced salidroside and tyrosol^22^. Nonetheless, TAT branch pathway is an important pathway to synthesize salidroside and tyrosol in natural *R. crenulata* uninfected with fungus^37^. This result indicated that *R. crenulata* would select optimum metabolic pathway or its combination according to special spatiotemporal conditions, and change varied with different fungal species. Similar examples supported this natural phenomenon. The ergot alkaloid synthesis pathway in *Lolium arundinaceum* induced with endophytic fungus *Epichloë* sp. has been studied extensively for the past two decades^38^. Currently, the content and type of ergot alkaloid in *L. arundinaceum* could be mediated through infection with genetically modified endophytic fungus *Epichloë* sp.; therefore, host plant would change the metabolic pathway in response to endophytic fungus infection^39^. Furthermore, a RNAi-based approach of reducing or increasing the concentration of mRNA from single gene could change alkaloid flux, in which the gene is related with endophytic fungus *Epichloë typhina* ^40^.

## Conclusion

Endophytic fungus plays important role in secondary metabolite accumulation in plant. Special endophytic fungus exhibits distinct promotion of particular secondary metabolite accumulation in a particular plant. In this study, endophytic fungus ZPRs-R11, that is, *Trimmatostroma* sp., was first proven to induce NO, SA, and H_2_O_2_ increase. This fungus subsequently induced gene to change the expression activities of some key enzyme genes, which resulted in secondary metabolite accumulation in *Rhodiola* plant. The use of exogenous donor of NO, SA, and H_2_O_2_ and their scavenging or inhibiting agents indicated the absence of interaction among NO, SA, and H_2_O_2_ and their involvement in fungus induced-salidroside and tyrosol production. Analysis results of key enzyme gene expression activity stated that *R. crenulata* can select particular synthesis pathway to respond to *Trimmatostroma* sp. and produce the bioactive product salidroside and tyrosol. This study will help further understand the interaction mechanism between endophyte and plant and provide scientific basis to improve the quality of *R. crenulata* and industrialization of salidroside and tyrosol.

## Materials and Methods

### Plant materials and treatments

Meristem cultures were established from *R. crenulata* explants (5,007 m, E92°22′31″–92°24′88″, N29°44′33″–29°46′56″) (Supplementary Fig. S1) according to the method of György *et al*.^41^. The explants were surface sterilized (75% ethanol, 30 s; washing with sterile water, three times; treatment with 0.1% HgCl_2_, 3–5 min) and grown in modified Murshige and Skoog (MS) medium supplemented with 0.5 mg L^−1^ thidiazuron, 0.5 mg L^−1^ naphthaleneacetic acid, 30 g L^−1^ sucrose, and 8 g L^−1^ agar in 300 mL tissue culture vessels. Rooting medium contained 0.5 mg L^−1^ indole butyric acid, 30 g L^−1^, sucrose and 8 g L^−1^ agar. All media pH were adjusted to 5.7–5.8 before autoclaving at 121 °C for 20 min. Plants were cultured in a climatron at 28 °C/22 °C day/night with a 14 h/10 h cycle, 30 µmol·m^−2^·s^−1^ light intensity, and 70–75% humidity and subcultured every 30 days (Supplementary Fig. S1).

### Endophytic fungus culture, inoculation, and reisolation

The endophytic fungus ZPRs-R11 (*Trimmatostroma* sp., GenBank No. KJ542345), isolated from *R. sachalinensis*, was subcultured with potato dextrose agar medium at 25 °C in the dark. ZPRs-R11 mycelian disks (5 mm) were obtained and placed near the rooting plantlet caudexes of seven-day-old *R. crenulata*. Equal-sized PDA disks were set as control. The tested plantlets were collected every two days. The test cycle was 20 days, and each experiment was performed in triplicate. The fungus ZPRs-R11 was re-identified to verify authenticity through morphological characteristics and 18S-rDNA-ITS sequence (Supplementary Fig. S1).

### H_2_O_2_ measurement

H_2_O_2_ was measured by modified titanium sulfate method^42^. Leaf samples (1 g) were ground with 5 mL of cold acetone in ice bath. The homogenate was centrifuged at 10,000 g for 20 min, and 2 mL of supernatant and 0.5 mL of 5% titanium sulfate were completely mixed fully. Afterward, 2 mL of strong ammonia water was added in the mixture, which was subsequently homogenized. The mixture was centrifuged at 10,000 g for 15 min. The supernatant was removed, and the sediment was dissolved with 5 mL of concentrated sulfuric acid (2 M) The H_2_O_2_ value was calculated through the H_2_O_2_ external standard at 415 nm with spectrophotometer (UV2700, Shimadzu, Japan).

### NO measurement

*R. crenulata* leaves (1 g) were uniformly ground with 3 mL of distilled water, aided with little silica sand. The mixture was centrifuged at 4,000 g for 20 min. Approximately 1 mL of supernatant was uniformly mixed with 1 mL of Greiss reagent (Sigma-Aldrich, USA) and incubated at 25 °C for 30 min. The NO content was measured on the basis of a standard NaNO_2_ curve at 550 nm according to modified Greiss reagent method^41^.

### SA measurement

SA quantification was performed using RP-HPLC (Agilent 1200, Agilent technology, USA) equipped with Thermo-C18 column (250 mm × 4.6 mm, 5 µm) and fluorescence detector under 294 nm of excitation wavelength and 426 nm of emission wavelength. The injection volume was 10 µL, and flow rate was 0.5 mL min^−1^ at 25 °C. The mobile phase was sodium acetate buffer (0.2 M):methanol (9:1 v/v).

The SA extraction method was modified according to that of Yuan *et al*.^16^. Leaf samples (1 g) were ground in liquid nitrogen and extracted ultrasonically in 1 mL of methanol. After centrifugation at 10,000 g for 15 min, the residue was re-extracted two times as above. The supernatants were combined and freeze dried. The concentrate was dissolved in 0.5 mL of trichloroacetic acid. After oscillation for 2 min, the mixture was extracted twice with 0.8 mL of acetic acid ester:cyclohexane (1:1 v/v). Organic phases were combined and freeze dried. The concentrate was dissolved with 0.6 mL of HPLC mobile phase and filtered with 0.22 µm Millipore membrane for detection.

### Treatment of plantlets with inhibitors and exogenous H_2_O_2_, NO, and SA

Sodium nitroprusside (SNP) was used as a NO donor. H_2_O_2_, SNP, and SA were obtained from Sigma–Aldrich (St. Louis, MO, USA). The specific scavenging or inhibiting agents of H_2_O_2_, NO, and SA were catalase (CAT), 2-(4-carboxyphenyl)-4,4,5,5-tetramethylimidazoline-1-oxyl-3-oxide potassium salt (cPTIO), and trans-cinnamic acid (CA), respectively. All of these agents were purchased from Sigma–Aldrich (St. Louis, MO, USA). All the exogenous donors and inhibitors were dissolved with ultrapure water. According to preliminary test results, the most suitable working concentration of SNP, SA, H_2_O_2_, and CA was 1 mM. Moreover, the most suitable concentrations of cPTIO and CAT to evaluate the interaction mechanism of three signaling pathways in this experiment were 30 μM and 2 mkat L^−1^, respectively. All the above solutions were filtered through 0.22 μm Millipore filter and sprayed directly on plant explant tissues. Scavenger or inhibitor was applied on plant at one day before ZPRS-R11 inoculation or spraying of exogenous donors. Ultrapure water was set as control.

### Expression activities of key enzyme genes

To examine the influence of endophytic fungi on the expression of key enzyme genes in *R. crenulata*, two constitutively expressed genes, namely, phytochelatin synthase (*PCS*) and ubiquitin (*UBQ*), were used as double-reference-genes. The 2^−ΔΔCT^ method^43^ was performed to relatively quantify the seven key enzyme genes in the biosynthesis pathway of tyrosol and salidroside; these genes included *PAL*, cinnamic-4-hydroxylase (*C4H*), *TYDC*, *TAT*, monoamine oxidase (*MAOA*), pyruvate decarboxylase (*PCD*), and UDP-glucosyltransferase (*UDPGT*). The primers of these genes are listed in Supplementary Table S1. Total RNA from the tissue-cultured plantlets of *R. crenulata* was isolated using Plant RNA Extraction Kit (Sangon Biotech., Shanghai, China). Subsequently, the first-strand cDNA was synthesized from 500 ng of total RNA in a volume of 20 µL using the TransScrip® One-Step gDNA remover and cDNA Synthsis SuperMix (TransGen. Biotech., Beijing, China) according to manufacturer’s protocol. All final cDNA samples were diluted 20-fold for subsequent quantitative real-time PCR (RT-PCR) reactions with quantitative RT-PCR reactions (CFX96TM Real-Time Detection System, Bio-Rad Laboratories Corp., USA) with SYBR Green QPCR Master Mix (TransGenBiotech). Each reaction (final volume of 15 µL) contained 2 µL of diluted cDNA, 7.5 µ of 2 × TransStart® Tip Green qPCR SuperMix, and 0.3 µL of each primer. The PCR conditions were as follows: 94 °C for 30 s, followed by 45 cycles of 94 °C for 5 s, 56 °C for 15 s, and 72 °C for 10 s.

### Extraction, identification, and qualification of salidroside and tyrosol

Dried plant powder (0.5 g) was sonicated for 30 min in 10 mL of methanol and re-extracted for another two times from the residue. After centrifugation at 5,000 g for 10 min, the combined supernatants were rotary evaporated at 50 °C. Methanol was added to a constant volume of 1 mL. The mixture was filtered with 0.22 µm Millipore membrane for qualitation and quantification.

Identification of salidroside and tyrosol was based on ^1^H-NMR and UPLC-Q/TOF/-MS according to our previous methods^44^. In brief, dried plant extracts were dissolved in methanol-*d*_4_ (CD3OD) to elucidate the structure through comparison of parameters, such as the chemical shift of standard salidroside and tyrosol (bought from National Institute for Food and Drug Control, Beijing), using an AVANCE 300 MHz spectrometer (Bruker, Switzerland). Molecular masses of salidroside and tyrosol were confirmed through comparison of parameters with the standard based on Waters Xevo G2Q-TOF (Micromass MS Tech., UK). The detection of salidroside and tyrosol content was conducted with HPLC (Agilent 1200, Agilent Tech., USA) equipped with a diode array detector and a Thermo-C18 column (250 mm × 4.6 mm, 5 µm) at 30 °C. The mobile phase was methanol: H_2_O_2_ (32:68 v/v) with a flow rate of 0.8 µL min^−1^. The monitoring wavelength was 277 nm.

### Statistical analyses

Data were edited with Microsoft Office Excel 2007 and Origin 8.0 (OriginLab Corp. US). The values are expressed as mean ± standard deviation. Statistical significance was evaluated using one-way ANOVA. Duncan’s multiple range tests were performed using SPSS 16.0 software (SPSS version 16.0, SPSS, Chicago, IL, USA). Correlation analysis was based on Pearson’s coefficient. Differences among means were identified significant at *P* < 0.05. Statistical evaluation on two means was performed by student’s *t*-test.

## Electronic supplementary material

Supplementary Table S1; Supplementary Figure S1; Supplementary Figure S2
