# Supplementary material for: Fungal endophyte-induced salidroside and tyrosol biosynthesis combined with signal cross-talk and the mechanism of enzyme gene expression in Rhodiola crenulata
Source: Sci Rep. 2017 Oct 2;7:12540. doi: 10.1038/s41598-017-12895-2 (PMC5624951; doi:10.1038/s41598-017-12895-2)
Supplement: Supplementary file 1 — Supplementary Table S1; Supplementary Figure S1; Supplementary Figure S2 [file 41598_2017_12895_MOESM1_ESM.pdf]

Fungal endophyte-induced salidroside and tyrosol biosynthesis combined with signal cross-talk and the mechanism of enzyme gene expression in *Rhodiola crenulata*

Jin-Long Cui[1], Ya-Nan Wang[1,2], Jin Jiao[1], Yi Gong[1,2] Jun-Hong Wang[1] & Meng-Liang Wang[1]

1. Institute of Applied Chemistry, Shanxi University, Taiyuan 030006, China.
2. Institute of Biotechnology, Shanxi University, Taiyuan 030006, China.

Correspondence and requests for materials should be addressed to J.L.C. (email: cjl717@sxu.edu.cn) or M.L.W. (email: mlwang@sxu.edu.cn)

Address: No.92, Wucheng Road, Xiaodian District, Taiyuan, 030006, Shanxi province, China
Tel: +86-351-7016101
Fax: +86-351-7016101

Supplementary Table S1 Primers of two constitutively expressed genes and seven key enzyme genes in *R. crenulata* used for quantitative real-time PCR.

**Gene name**	**Gene**	**primer (5'–3')**	**Amplicon size (bp)**
Phenylalanine ammonia-lyase	*PAL*	F:AGACCTGCTCAAAGTCGTCG	143
		R:TCGGGGTCTTCTCGTTTTCG	
Cinnamic-4-hydroxylase	*C4H*	F:GTTCAGACCCGAGAGGTTCC	83
		R:CCACACCGAACGGCAAATAC	
Phytochelatin Synthase	*PCS*	F: TGAGGTGGCGACTGATAACC	130
		R: GGGAGAACCCTCCGGTACAA	
Ubiquitin	*UBQ*	F: GCGCCACAGATTGATTCGTC	147
		R: CTTCAGCGCCCATTT TCCAC	
Tyrosine decarboxylase	*TYDC*	F: GCAGAGTCGTCTCGAGTGTC	98
		R: GTCCTTCTGTATGTCCGCCC	
Monoamine oxidase	*MAOA*	F:CCCATCAAGACGAGGGCTAC	94
		R:ACGACCAGCTTCGTAAGTCC	
Tyrosine transaminase	*TAT*	F: AACGTGTGGTCGTCCTAAGC	146
		R: TTGCTGTTGTCTCTCCGCTC	
Pyruvate decarboxylase	*PCD*	F:AGAAGAGTTGACCCGCAAAG	100
		R:GCCATTGAACCACGAATCTC	
UDP-glucosyltransferase	*UDPGT*	F:ACGGCTTACTGGCTCAACTC	82
		R:ACGACATGGGAGGCGTAAAG	

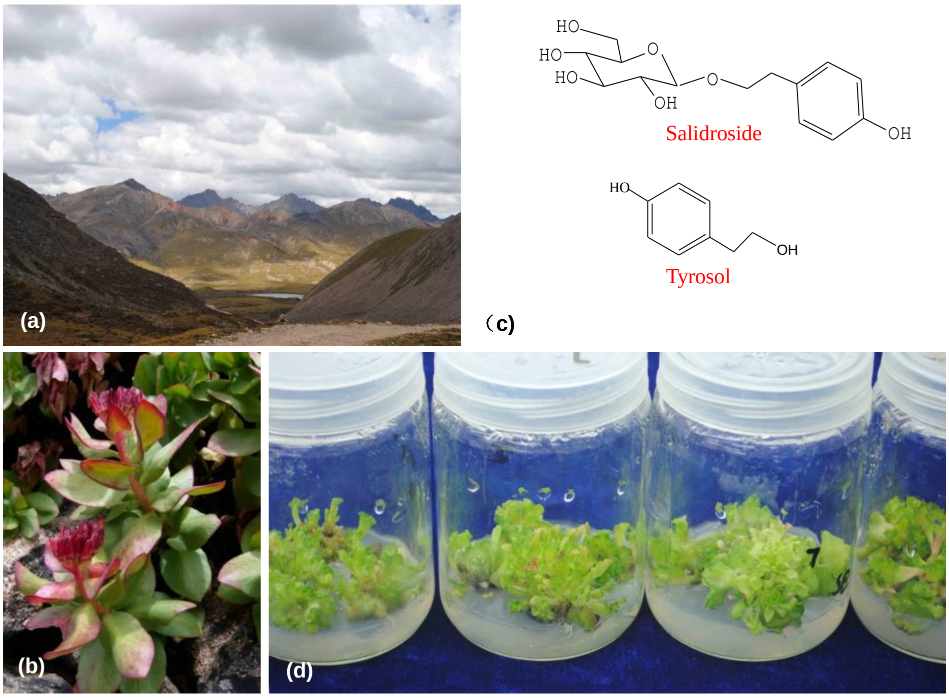

Supplementary Figure S1: Mountains with an altitude of more than 3500 m (**a**) are inhabited with wild *Rhodiola crenulata* (**b**) at Qinghai–Tibet plateau in China; such are is characterized with dryness, oxygen deficiency, and intense ultraviolet radiation. Salidroside and tyrosol (**c**) are two of the most effective pharmacological components in adaptogenic plant *R. crenulata*, which is difficult to culture under low-altitude environment. However, tissue cultured plantlets (**d**) were obtained successfully from the bud of *R. crenulata* in our laboratory. These photographs in figures S1 were taken by Dr. Jinlong Cui.

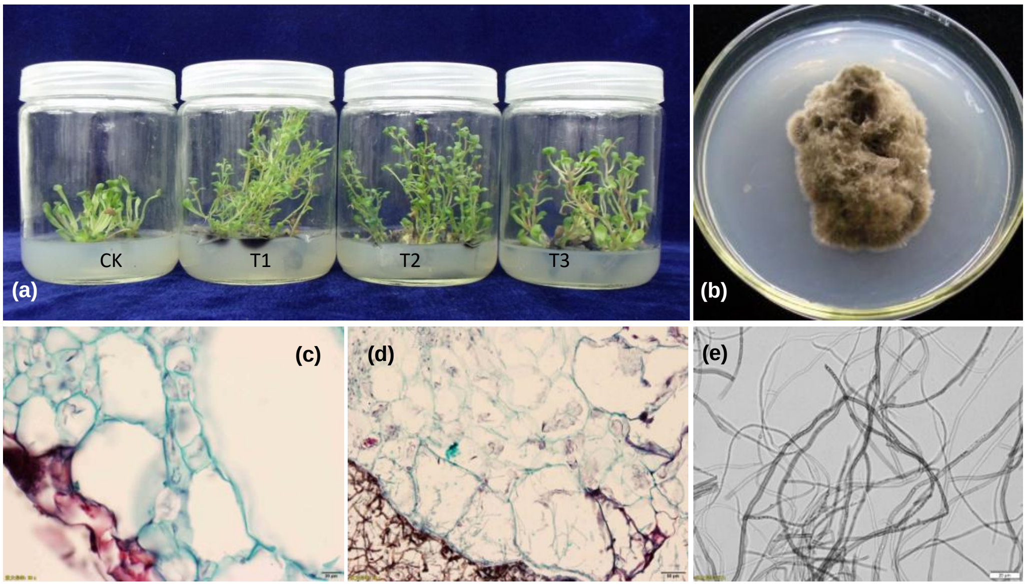

Supplementary Figure S2: *R. crenulata* plantlet inoculated with endophytic fungus Rs-R11 (**a**). The fungal invasion was investigated using microtome technology (**b**). No hyphae were observed in control (**c**). Hyphae gradually invaded and created symbiosis relationship with tissue and cell in plantlets (**d**). The fungus was re-isolated, identified, and confirmed in plantlet through its colony (**b**), microscopic characteristic (**e**), and rDNA ITS sequence according to the sequence of KJ542345 in GenBank. These photographs in figures S2 were taken by Dr. Jinlong Cui.
